# A Review of Antimicrobial Mechanisms of Graphene Oxide, Reduced Graphene Oxide, and Graphene Nanoplatelets: Implications for Polymer-Based Dental Materials

**DOI:** 10.3390/nano16211342

**Published:** 2026-10-23

**Authors:** Inês Côrte-Real, Driss Belayach, Ricardo Martuci, João Sampaio-Fernandes, Maria Helena Figueiral

**Affiliations:** 1 Faculdade de Medicina Dentária, Universidade do Porto, Rua Dr. Manuel Pereira da Silva, 4200-393 Porto, Portugal; indust.engineering@gmail.com (D.B.); jfernandes@fmd.up.pt (J.S.-F.); mhsilva@fmd.up.pt (M.H.F.); 2 Instituto de Ciência e Inovação em Engenharia Mecânica e Engenharia Industrial (INEGI), Universidade do Porto, Campus da FEUP, R. Dr. Roberto Frias 400, 4200-465 Porto, Portugal

**Keywords:** graphene oxide, reduced graphene oxide, graphene nanoplatelets, carbon nanomaterials, antimicrobial mechanisms, dental biomaterials

## Abstract

Graphene-based materials, including graphene oxide (GO), reduced graphene oxide (rGO), and graphene nanoplatelets (GNPs), exhibit a relatively large surface area and tunable surface chemistry, enabling diverse biological interactions. These interactions depend on oxygen-containing functional groups, oxidation degree, lateral size, and surface charge. In dental materials, these properties support incorporation of graphene-based materials into polymer matrices to reduce microbial adhesion, inhibit biofilm formation, and limit infection-related degradation. This review critically evaluates GO, rGO, and GNPs in polymer-based dental materials, focusing on structure–property–biological response relationships and antimicrobial performance. Current evidence indicates that GO demonstrates the most consistent antibacterial and antifungal activity, mainly due to its high density of functional groups and hydrophilicity. In contrast, rGO and GNPs show more variable or moderate antimicrobial effects, often requiring surface modification, optimized composite design, or higher material loading. Here, key limitations affecting clinical application are also discussed. Several biological effects are concentration-dependent, including human cytotoxicity, host enzyme interactions, potential immune responses, long-term biocompatibility, and stability under oral conditions. By integrating mechanistic insights across graphene-based materials, this review clarifies their potential and current limitations for polymeric dental applications and identifies priorities supporting the transition from laboratory-scale research to clinical and industrial implementation strategies.

## 1. Introduction

Microbial adhesion and biofilm formation remain important challenges in dentistry because they can compromise the performance of dental biomaterials and contribute to secondary caries, prosthetic failure, peri-implant diseases, and chronic oral inflammation. Bacteria and fungi are relevant microorganisms in oral biofilm-associated diseases. In the context of graphene-modified dental polymers, representative species investigated include *Streptococcus mutans* and *Candida albicans*, which are associated with dental caries and denture-related stomatitis, respectively [1]. In recent years, graphene-based nanomaterials have attracted increasing attention as potential antimicrobial agents because of their distinctive physicochemical properties [2,3,4]. In restorative and prosthetic materials, including polymer-based systems, surface characteristics and material composition may influence microbial adhesion, biofilm development, and biological responses at the material–tissue interface [5,6,7]. Surface roughness, wettability, surface energy, surface charge, and protein adsorption can influence initial microbial adhesion and subsequent biofilm development, although their effects depend on the material and oral environment [8,9].

Nevertheless, direct comparisons of the antimicrobial behaviour of different graphene-based materials, both in isolation and when incorporated into polymer matrices, remain fragmented [1,10,11]. Variability in synthesis methods, physicochemical characteristics, and experimental conditions further complicates the interpretation and translation of existing findings, particularly in dental polymer applications.

Several approaches have been investigated to limit microbial contamination in dentistry, including antibiotics, conventional antimicrobial agents, metal-based nanomaterials, naturally derived compounds, and functional nanostructures. These approaches differ in their mechanisms of action, delivery strategies, effects on material properties, and potential limitations. Metal–organic frameworks (MOFs), for example, have attracted interest as functional platforms for antimicrobial applications and therapeutic delivery in dentistry [12]. Hybrid materials have also been explored, including phosphazene-modified methacrylic compositions for three-dimensional (3D) printing of dental crowns, for which antibacterial effects against *Streptococcus mutans* have been reported under the conditions tested [13]. These approaches illustrate the diversity of strategies available for developing antimicrobial dental materials. Beyond graphene oxide, reduced graphene oxide, and graphene nanoplatelets, other carbon-based nanomaterials have also been investigated for antimicrobial applications. For example, gamma-ray-modified carbon quantum dots have demonstrated antibacterial activity, while graphene quantum dots incorporated into bacterial cellulose hydrogels have shown light-activated antibiofilm effects associated with singlet oxygen production [14,15]. However, their results cannot be directly compared without considering differences in composition, experimental design, target microorganisms, and outcome measures. Against this background, graphene-based materials warrant investigation because their physicochemical characteristics can be modified and tailored to specific applications, although their antimicrobial performance and safety must be assessed for each formulation.

Graphene-based materials have been investigated for their interactions with microbial cells and biological components, with several mechanisms proposed to explain their antimicrobial effects. These include physical interactions with microbial membranes and oxidative stress-related effects [16,17,18]. Other studies have explored the contribution of surface chemistry and electron-transfer processes to the biological activity of these materials [19,20]. However, the relative contribution of individual mechanisms is not necessarily uniform across different graphene derivatives or experimental systems. Lateral dimensions, oxidation state, surface functional groups, concentration, dispersion, and exposure conditions may influence the observed biological response [21,22,23,24]. Further physicochemical and biological factors may also affect the interpretation of experimental findings [25,26,27]. Consequently, antimicrobial activity should be interpreted in relation to the specific characteristics of the material tested rather than attributed to graphene-based materials as a homogeneous class.

The present review focuses on three graphene-based materials: graphene oxide (GO), reduced graphene oxide (rGO), and graphene nanoplatelets (GNPs). These materials differ in structure and surface chemistry, providing a basis for examining how material-specific characteristics may influence antimicrobial behaviour and incorporation into polymer-based dental systems. GO contains oxygen-bearing functional groups that influence its surface chemistry and interactions with aqueous and biological environments. Reduction of GO produces rGO with a lower oxygen content and a partially restored graphitic structure [16,28]. In contrast, GNPs generally consist of stacked graphene sheets and are investigated, among other purposes, as reinforcing additives in composite materials [29,30,31]. These differences are relevant to interpreting antimicrobial findings and selecting an appropriate graphene-based additive for a given dental application. Nevertheless, the biological and material-related effects of each form depend on its actual physicochemical characteristics, formulation, concentration, and experimental conditions.

Experimental studies have investigated graphene-based materials in dental adhesive systems, restorative composites, dentin-related applications, and acrylic or other polymer-based materials [3,4,7,10]. Antimicrobial effects have been reported for particular formulations, including graphene nanoplatelet-containing dental resins and graphene-modified polymethylmethacrylate [1]. Studies involving rGO-modified acrylic resins have also examined microbiological and mechanical outcomes [11]. These findings support continued investigation of graphene-based additives in dental materials. However, they do not establish consistent antimicrobial effects across all formulations or demonstrate that laboratory observations necessarily translate into clinical benefits. Interpretation requires consideration of the graphene derivative used, its concentration and distribution within the matrix, the material’s surface properties, the microorganisms tested, and the experimental methods applied.

Several previous reviews have addressed the biomedical applications and physicochemical characteristics of graphene-based materials [32,33,34]. Other reviews have examined antimicrobial mechanisms, biological interactions, and safety considerations [5,6,10,35,36,37]. More specifically, reviews of graphene-based antimicrobial systems have discussed proposed mechanisms and factors influencing antimicrobial performance [17,20,29]. Dental reviews have also considered a wider range of applications, including restorative materials, tissue-related applications, and other therapeutic approaches [2,6,10]. In addition, Martuci et al. recently published a scoping review mapping the antimicrobial effects of different graphene-based materials in dental biomaterials and describing the available evidence on antimicrobial effectiveness and biological responses [38]. This work is particularly relevant when defining the scope and contribution of the present review.

To ensure terminological consistency and define the scope of the present review, the term “graphene-based materials” refers specifically to graphene oxide (GO), reduced graphene oxide (rGO), and graphene nanoplatelets (GNPs) obtained by chemical synthesis or processing using standard methods and characterized using techniques such as Raman spectroscopy, X-ray photoelectron spectroscopy (XPS), or transmission electron microscopy. Materials obtained primarily by thermal decomposition or carbonization and lacking structural and spectroscopic confirmation of graphene-like properties are not considered graphene-based materials within the scope of this review.

However, the available evidence remains difficult to compare because of differences in synthesis methods, physicochemical characterization, experimental protocols, and the dispersion and interfacial behaviour of graphene-based materials within polymer matrices [11,22,31]. Furthermore, the predominance of laboratory-based evidence and the limited assessment of performance under simulated intraoral and in vivo conditions leave unresolved whether antimicrobial benefits can be achieved without compromising mechanical properties, aesthetic outcomes, and long-term biocompatibility [22]. Accordingly, this review aims to critically compare graphene oxide (GO), reduced graphene oxide (rGO), and graphene nanoplatelets (GNPs) in terms of their physicochemical characteristics, antibacterial and antifungal activities, and proposed mechanisms of action, with particular emphasis on how material-specific properties may influence antimicrobial effects. It further evaluates the relevance of these mechanisms to polymer-based dental materials, considering the reported antimicrobial outcomes alongside potential effects on material properties, aesthetic outcomes, cytotoxicity, and biocompatibility. Unlike broader reviews addressing the biomedical applications or antimicrobial activity of graphene-based materials across different fields, the present review integrates material-specific characteristics, proposed antibacterial and antifungal mechanisms, and experimental findings from polymer-based dental systems within a common interpretative framework. It also considers limitations affecting the interpretation and clinical relevance of the available evidence. By distinguishing reported findings from proposed mechanisms and identifying gaps in the evidence, this review aims to clarify the current knowledge and highlight research priorities for the further evaluation of graphene-based materials in dentistry.

## 2. Graphene-Based Material: Structure, Physicochemical Properties and Antimicrobial Mechanisms of Action

Graphene consists of a single-layer nanosheet of carbon atoms arranged in a two-dimensional hexagonal honeycomb lattice, representing one of several carbon allotropes whose structural diversity arises from strong covalent bonding [39]. Its distinctive structural and physicochemical properties have motivated its investigation for applications in electronics and nanotechnology, as well as in biomedical materials [32,33]. In dentistry, graphene-based materials have been explored as potential functional additives for dental biomaterials [2]. However, their biological effects, including antimicrobial activity and biocompatibility, depend on their physicochemical characteristics and experimental conditions [21,40].

First isolated in 2004 by Novoselov et al. [41], graphene has since given rise to numerous derivatives, including graphene oxide (GO), reduced graphene oxide (rGO), graphene nanoplatelets (GNPs), graphene quantum dots (GQDs), graphene nanoribbons (GNRs), and hybrid nanocomposites. GO, rGO, and GNPs have been investigated as functional additives in polymer-based dental materials, with reported antimicrobial effects that vary with the graphene-based material, its concentration, and the composite formulation [3,4]. However, differences in experimental designs and outcome measures preclude a general ranking of their antimicrobial efficacy.

Graphene quantum dots (GQDs) and graphene nanoribbons (GNRs) have also been investigated for antimicrobial applications. For example, graphene oxide quantum dots (GOQDs) have been incorporated into electrospun poly(ether sulfone) nanofibrous membranes, which exhibited antibacterial activity against *Escherichia coli*, *Staphylococcus aureus*, and *Bacillus cereus* [42]. In a separate study, a hybrid nanocomposite comprising graphene nanoribbons and chitosan-coated silver nanoparticles exhibited enhanced antibacterial activity under near-infrared irradiation, attributed to a photothermal effect [43]. These findings demonstrate the antimicrobial potential of specific GQD- and GNR-based systems in non-dental applications. However, they do not establish their efficacy in polymer-based dental materials or demonstrate that surface functionalization or hybridization is generally required for antimicrobial activity. Accordingly, the present review focuses on GO, rGO, and GNPs, for which the subsequent sections discuss evidence relevant to polymer-based dental materials.

The term “graphene-based materials” as used herein refers specifically to graphene oxide (GO), reduced graphene oxide (rGO), and graphene nanoplatelets (GNPs) that have been chemically synthesized or processed using established methods and characterized by techniques such as Raman spectroscopy, X-ray photoelectron spectroscopy (XPS), or transmission electron microscopy. Materials resulting primarily from thermal decomposition or carbonization processes that lack structural and spectroscopic confirmation of graphene-related features are not considered graphene-based materials within the scope of this review.

GO, rGO, and GNPs are most widely utilized for material functionalization. In dental biomaterials, these graphene forms are increasingly explored as functional additives in polymeric matrices to mitigate microbial adhesion, biofilm formation, and infection-related degradation [3,4,34]. GO contains oxygen-containing functional groups that contribute to its surface polarity and aqueous dispersibility [10]. The properties of rGO differ from those of GO because of its lower oxygen content and partially restored graphitic structure [6]. Both materials have been investigated in biomedical applications, including drug delivery, tissue engineering, and dental materials [2,6,10].

**Figure 1:**
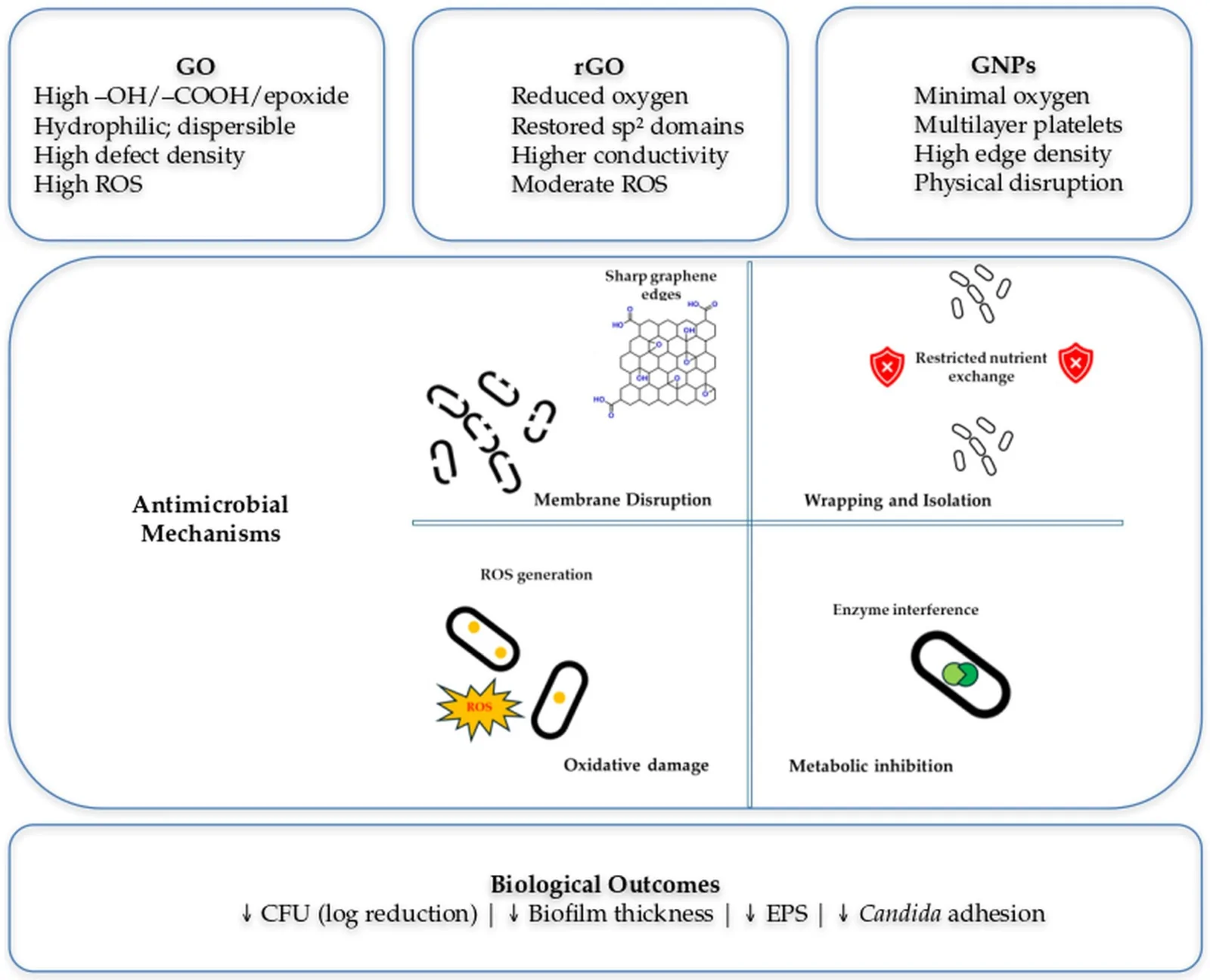
Structure–property–mechanism of action framework of GO, rGO, and GNPs. Proposed antimicrobial mechanisms and biological outcomes. ↓ indicates a decrease; CFU, colony-forming units; EPS, extracellular polymeric substances.

Although GO and rGO display antimicrobial activity, their mechanisms of action and overall effectiveness differ due to variations in surface chemistry, degree of oxidation, and interactions with microbial membranes [34,35]. GO has been associated with physical disruption of microbial membranes [34,44]. Oxidative damage involving reactive oxygen species (ROS) has also been proposed as a contributor to its antimicrobial activity [3,44]. Other reported effects include interference with cellular or metabolic processes (Figure 1) [44].

GO can also modulate key biological pathways, including gene expression, enzyme activity, and quorum sensing. Its high density of oxygen-containing functional groups (e.g., hydroxyl, epoxy, carbonyl, and carboxyl) promotes strong interfacial interactions with biomacromolecules. Within a Hard and Soft Acids and Bases (HSAB) framework, these oxygenated moieties may be considered in terms of their interactions with acidic sites in biological components [45,46]. In parallel, electron donor–acceptor (EDA) interactions and interfacial charge-transfer processes may contribute to adsorption phenomena and redox activity [16,17]. These proposed interactions may be relevant to the generation of oxidative stress at biological interfaces [47].

Furthermore, these functional groups enable synergistic interactions with conventional antimicrobial agents by facilitating drug loading or conjugation, thereby supporting targeted and controlled release [18,19,45,46]. As a result, GO has demonstrated antimicrobial efficacy, particularly against bacteria and fungi, when appropriately functionalized [16,45,46].

In contrast, rGO, characterized by a reduced content of oxygen-containing groups and a more reconstituted graphene lattice, exhibits higher electrical conductivity but lower hydrophilicity, which may diminish antimicrobial performance in certain contexts. The partial restoration of sp^2^ carbon domains increases surface π–electron density and reduces polarity, potentially favoring hydrophobic contact, π–π interactions, and membrane-associated mechanisms over interactions dominated by polar surface functionalities. Nonetheless, rGO retains notable bactericidal activity, particularly through mechanisms involving physical disruption of microbial membranes (Figure 1) [16,20,28,48].

GNPs constitute a structurally distinct class of graphene-based materials, consisting of stacks of few-layer graphene sheets with large lateral dimensions and thicknesses ranging from a few layers (~0.34 nm) to several tens of nanometers [29]. Although GNPs contain fewer oxygen-containing functional groups than GO or rGO, reducing the contribution of oxygen-mediated HSAB-type and surface redox-related EDA interaction, they retain excellent mechanical strength, thermal stability, and electrical conductivity [17,29]. These properties make them particularly suitable for composite reinforcement, electronic devices, and structural applications.

**Figure 2:**
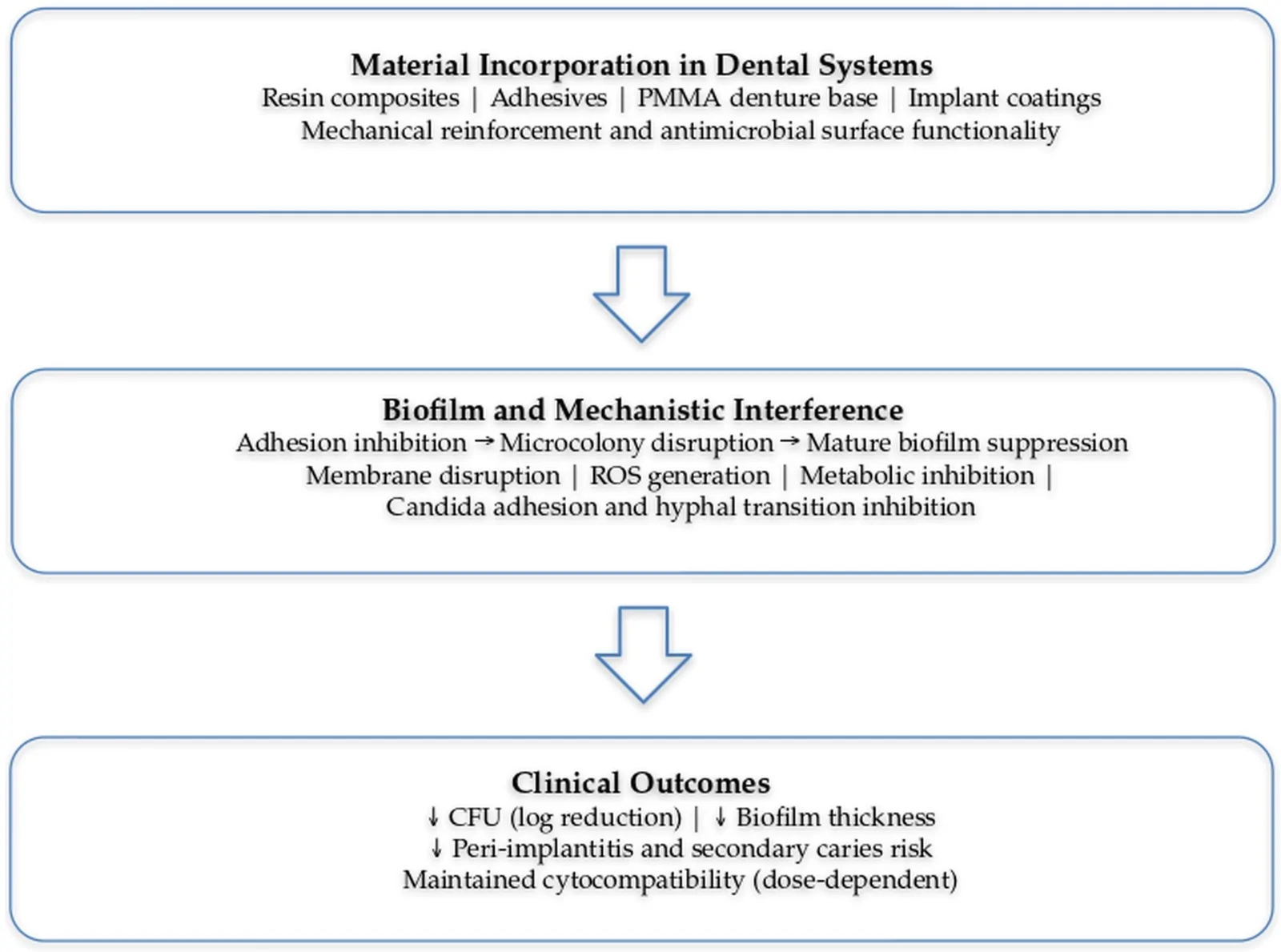
Integration of graphene-based materials into dental biomaterials and their antimicrobial mechanisms of action, resulting in reduced biofilm formation and microbial viability while maintaining cytocompatibility. The downward arrow (↓) indicates a decrease.

Their extensive surface area and layered morphology also contribute to moderate antimicrobial activity, primarily through the physical disruption of microbial membranes (Figure 1). However, their larger size and comparatively lower hydrophilicity may reduce direct microbial contact. Consequently, GNPs are frequently functionalized or incorporated into hybrid nanocomposites to enhance their antimicrobial performance [29,49]. Interference with enzymatic activity may contribute to the antimicrobial effects of graphene-based materials. GO-induced oxidative stress and rGO-mediated membrane disruption, together with possible interference with key metabolic enzymes, may significantly impair microbial viability [30,50,51]. Although GNPs exhibit lower surface reactivity, steric or charge-related interactions may still disrupt enzymatic pathways, particularly when functionalized [52,53]. Antimicrobial mechanisms such as oxidative damage and membrane disruption by graphene-based materials are strongly dependent on material-specific parameters, including particle size, degree of oxidation, aggregation, surface functionalization, and applied dose. These mechanisms should therefore not be interpreted as universally applicable across different graphene-based materials or dental polymer systems [44,48]. Figure 2 summarizes the proposed antibacterial mechanisms and outcomes.

The distinct physicochemical properties of graphene-based materials make the selection of GO, rGO, or GNPs particularly critical for biomedical dental material applications. (Table 1). However, as discussed in Section 3, GO’s, rGO’s, and GNPs’ antibacterial and antifungal performance and safety considerations must be carefully evaluated.

Despite the extensive investigation of graphene-based nanomaterials, direct comparisons of the antimicrobial behavior of different graphene-based materials remain fragmented across polymeric and non-polymeric systems. Recent reviews likewise highlight the influence of material characteristics and experimental conditions on antimicrobial performance, reinforcing the need for cautious interpretation of cross-study comparisons [48,54].

Owing to its high density of oxygen-containing functional groups, GO is readily functionalized and can often be employed at lower concentrations, thereby improving biocompatibility while maintaining robust antimicrobial efficacy when incorporated into polymeric matrices [34,35]. In contrast, rGO and GNPs typically require higher concentrations to achieve comparable functional outcomes, which may increase human cytotoxicity risks and restrict their biomedical applicability unless they are further functionalized or integrated into hybrid systems [29,49]. Moreover, the choice of graphene type in polymeric composites is strongly influenced by its surface chemistry and the nature of the polymer matrix. Hydrophilic polymers, such as polyvinyl alcohol (PVA), chitosan, and polyethylene glycol (PEG), generally interact more effectively with GO due to its high density of oxygen-containing functional groups and resultant surface polarity, whereas more hydrophobic polymers may exhibit better compatibility with rGO or GNPs [31]. The intended mode of incorporation (whether as a surface coating or bulk embedding) further affects the dispersion, functionalization potential, and interfacial interactions of the graphene within the polymer, which, in turn, influence both performance and safety [22]. Consequently, optimizing physicochemical compatibility between the selected graphene form and the polymer is essential to achieve desired functional outcomes, including mechanical reinforcement, biointerface formation, and cytocompatibility. The antimicrobial efficacy of graphene-based materials in polymer matrices is similarly governed by surface chemistry, morphology, and the ability to establish effective biointerfaces, with graphene-based materials often demonstrating superior antimicrobial performance compared to graphene-based materials alone [23,31].

Surface chemical characterization by XPS (Table 2) enables the assessment of carbon bonding states and oxygen-containing functional groups in graphene-based materials [16,24,25]. These data can help interpret differences in surface chemistry and potential interactions with biomolecules and microbial cell envelopes [26,27,30].

## 3. Antimicrobial Activity of Graphene-Based Materials

Graphene-based materials have been shown to exert harmful effects against diverse classes of organisms, including bacteria, fungi [45,46], viruses [55,56,57], and certain parasites [58]. However, as abovementioned, this review focuses specifically on the antibacterial and antifungal activities of GO, rGO, and GNPs as these are the graphene-based materials most extensively investigated for dental applications, and because bacteria and fungi represent the main oral pathogens. The antibacterial and antifungal responses to these graphene-based materials are modulated by their physicochemical attributes, such as surface functionalization, particle size, and degree of oxidation, which influence their interaction with microbial cells. Accordingly, the antimicrobial behavior of GO, rGO, and GNPs, is discussed in the following sections to illustrate their relevance and prominence in current dental-related research.

### 3.1. Antibacterial Activity of Graphene-Based Materials

Dental caries is a multifactorial pathology primarily associated with the activity of acidogenic bacteria within oral biofilms, which reduces the local pH and promotes the demineralization of enamel and dentin. Sustained acidic conditions progressively compromise the integrity of dental hard tissues, ultimately leading to cavitation. The antimicrobial potential of GO and rGO against key bacterial species implicated in this process, most notably *Streptococcus mutans* (*Strep. mutans*), has been demonstrated across multiple experimental studies as shown by reductions in colony forming units (CFU) of up to 98% in in vitro assays [7]. Selected examples are outlined below, organized according to the type of antibacterial performance observed.

#### 3.1.1. Antibacterial Performance and Remineralization/Protective Effects of GO-Based Composites Against *Strep. mutans*

Recent in vitro investigations [7] have demonstrated that functionalized GO may serve not only as an antimicrobial material but also as a protective agent capable of reinforcing mineral stability at the tooth surface. In the referenced study, functionalized GO formed a stable polymer-modified coating on dentin substrates, acting as a “mineral shield.” This layer established a persistent interface with ions present in the surrounding environment, compensating for mineral loss induced by acidic challenges and supporting ongoing remineralization processes. By enveloping the dentin surface and restricting acid diffusion, functionalized GO contributed to the maintenance of mineral homeostasis, thereby mitigating demineralization and helping preserve structural integrity. In the same study [7], five distinct GO functionalized nanocomposites were synthesized, GO–Ag, GO–Ag–CaF_2_, GO–CaF_2_, GO–Zn, and GO–Ca_3_(PO_4_)_2_, and applied as surface treatments to hydroxyapatite discs and human dentin specimens. Their performance was assessed in terms of antibacterial activity against *Strep. mutans*, dentin tubule sealing capacity, effects on tooth color, and cytotoxicity in cell culture conditions. GO–Ag and GO–Ag–CaF_2_ exhibited the highest percentage reduction in CFU with the GO–Ag–CaF_2_ formulation achieving a 98% reduction in CFU, surpassing the activity of silver diamine fluoride (Saforide). These formulations, however, induced a mild yellowish discoloration [59]. GO–CaF_2_ reduced CFU by ~10–20%, GO–Zn by ~15–30%, and GO–Ca_3_(PO_4_)_2_ by ~5–15% (Table 3) [7], lower than those of pure GO, but did not produce noticeable color alterations (Table 3) [7].

Though the qualitative descriptors of antibacterial activity and CFU percentage reduction in Table 3 do not provide quantitative data, they allow for comparison of the relative antibacterial efficacy of the different GO-based composites. GO–Ag and GO–Ag–CaF_2_ consistently demonstrated superior antibacterial performance against *Strep. mutans* across the tested concentration range, with GO–Ag–CaF_2_ exhibiting the strongest effect, indicative of a synergistic contribution of silver and calcium fluoride. In contrast, GO–CaF_2_, GO–Zn, and GO–Ca_3_(PO_4_)_2_ showed weaker and more concentration-dependent antibacterial activity, suggesting that the antimicrobial efficacy of GO-based composites is strongly influenced by the nature of the functionalizing agent.

To investigate the protective capacity of GO functionalized composites against dentin demineralization, dentin specimens were coated with different GO functionalized formulations and subsequently exposed to acidic conditions [7]. As an initial challenge, the samples were immersed in an ethylenediaminetetraacetic acid (EDTA) buffer, simulating the chelating and erosive effects commonly encountered during clinical procedures. Scanning electron microscopy (SEM) analyses confirmed that the GO functionalized treatments generated a thin surface film that partially or completely occluded dentinal tubules. Following EDTA exposure, tubule sealing was evident in dentin treated with GO–Ag, GO-Ag–CaF_2_, GO–CaF_2_, GO–Ca_3_(PO_4_)_2_, and GO–Zn, whereas Saforide-treated surfaces still displayed numerous open tubules despite its established clinical application. To further evaluate acid resistance, the same specimens were immersed in a citrate buffer (pH 6.0), representative of the mildly acidic environment characteristic of early carious lesions. SEM imaging demonstrated that the coatings produced by GO–Ag, GO–Ag–CaF_2_, GO–CaF_2_, and GO–Ca_3_(PO_4_)_2_, remained continuous and effectively maintained tubule occlusion under these conditions, indicating strong resilience to acid-induced degradation. In contrast, the GO–Zn coating showed compromised stability, with multiple tubules re-opening and evidence of intertubular surface deterioration. Saforide-treated dentin additionally presented a rough and irregular topography, likely attributable to aggregation of silver diamine fluoride particles upon acid exposure. Overall, these observations indicate that although Saforide offers some degree of protection, GO functionalized composites (particularly those functionalized with silver, calcium fluoride, or phosphate) produce more uniform and acid-resistant surface barriers. Their capacity to achieve consistent tubule sealing underscores their potential to reduce dentin hypersensitivity and limit demineralization in cariogenic environments.

Concentration-dependent effects of GO-based composites on bacterial metabolic activity were also assessed by XTT assay (Table 4) [7]. While the absorbance values are estimates from graphical data, the data reveal a consistent reduction in metabolic activity at higher concentrations for GO–Ag, GO–Ag–CaF_2_, and GO–Zn formulations, supporting their enhanced antimicrobial potency. Importantly, these trends suggest that antibacterial performance may be influenced by both concentration and the incorporation of metal-based components, while pure GO and GO–CaF_2_ exhibit comparatively limited inhibitory effects.

The findings reported in [7] indicate that functionalized GO formulations, notably GO–Ag, GO–Ag–CaF_2_, and GO–CaF_2_, exhibit a favorable combination of antibacterial activity, resistance to acid-induced degradation, and effective dentinal tubule occlusion. Among these, GO–CaF_2_ demonstrates the most suitable aesthetic profile, whereas GO–Ag and GO–Ag–CaF_2_ are associated with a slight yellowish tooth discoloration, which may restrict their application in areas of high aesthetic demand. All composites showed low cytotoxicity at lower concentrations, although mild cytotoxic effects emerged at 0.1% (*w*/*v*). The authors of this study emphasize the need for additional in vivo investigations to validate the biocompatibility and clinical applicability of these materials within preventive and restorative dentistry. It should be noted that the antibacterial data summarized in Table 3 and Table 4 are estimates from graphical data which limits direct numerical comparison but remains valuable for identifying performance trends among different GO-based formulations.

#### 3.1.2. Enhancement in Polymethyl Methacrylate (PMMA) Resin Antibacterial Activity by Combination with GNPs Targeting *Strep. mutans*

The incorporation of additional functional components into composite materials such as resins is essential to satisfy the complex performance demands of dental applications. In this context, graphene has attracted considerable interest due to above mentioned properties namely, the abundance of modifiable functional groups, ease of chemical tuning, exceptionally relatively high surface area and its intrinsic antimicrobial activity. These characteristics make graphene a valuable candidate for combination with other biomaterials and biomolecules in the development of next-generation dental technologies [60].

Polymethyl methacrylate (PMMA) is widely used in dentistry due to its favorable aesthetics, ease of processing, and cost-effectiveness. However, its susceptibility to microbial colonization and biofilm formation remains a clinical concern. Recent studies have therefore explored the incorporation of graphene-based nanomaterials into PMMA matrices to enhance antimicrobial performance, with promising results reported for graphene-reinforced PMMA composites [1]. Biofilm accumulation on the surface of dental prostheses can induce irritation of the adjacent oral mucosa, giving rise to erythema, discomfort, and inflammatory reactions collectively classified as denture-related stomatitis. To mitigate this problem Salgado et al. [1] investigated whether the antimicrobial performance of PMMA, a polymer widely used in denture bases, could be enhanced through the incorporation of GNPs, while maintaining the mechanical and functional integrity of the material. GNPs were incorporated into PMMA at concentrations of 0.01%, 0.1%, 0.25%, and 0.5% (wt), and the resulting 3D-printed composites were exposed to *Strep. mutans* cultures for 24 and 48 h to assess antibacterial effects. A marked reduction in CFU was observed after 48 h in samples containing 0.01%, 0.1%, and 0.25% GNPs. At 48 h, CFU decreased from ~12.8 log CFU/mL in control to ~7.8 log CFU/mL in 0.01% GNP, ~8.2 log CFU/mL in 0.1% GNP, and ~7.9 log CFU/mL in 0.25% GNP samples (Table 5) [1]. At the earlier 24 h time point, significant inhibition was detected only in the specimens incorporating the highest concentration (0.5% wt) (Table 5).

These results indicate that GNP incorporation produced concentration- and time-dependent changes in bacterial viability, with higher concentrations showing a more immediate antimicrobial effect. The authors highlight the need for additional human cytotoxicity assessments, particularly for the higher GNP concentrations, to ensure the biocompatibility of these graphene-modified PMMA composites for clinical use.

In a complementary experiment, Salgado et al. [1] incorporated GNPs into 3D-printed PMMA resins at concentrations of 0.01%, 0.1%, 0.25%, and 0.5% (*w*/*w*) to assess how these additions altered surface characteristics and, in turn, affected microbial adhesion. SEM analysis revealed that the inclusion of GNPs led to an increase in surface roughness, with roughness values rising proportionally to the GNPs concentration (Table 6).

Surface roughness (Ra) increased from 1.10 μm (control) to 1.80–1.90 μm in 0.25–0.5% (*w*/*w*) GNP samples (Table 6), yet CFU counts of *Strep. mutans* were reduced which aligns with established evidence [61,62]. Despite this increase in surface roughness, the formation of *Strep. mutans* biofilm was markedly reduced across the GNP-containing samples. These findings indicate that the antimicrobial activity imparted by GNPs was sufficiently potent to counteract the expected decrease in bacterial adhesion associated with rougher surfaces, thereby inhibiting both initial microbial attachment and subsequent biofilm development.

When considered collectively, the critical analysis of the experimental work of Salgado et al. supports the potential of GNP-enhanced PMMA as a promising next-generation dental biomaterial. By combining intrinsic antimicrobial properties with compatibility for 3D-printing workflows, these composites offer a strategic advantage for preventing prosthesis-associated oral infections and contribute to ongoing advancements in functional dental materials.

#### 3.1.3. Enhancement in Antibacterial Activity of rGO-Modified Acrylic Resins Against Oral Bacteria

To investigate the antimicrobial potential of rGO as a nanofiller in dental polymers, Sahm et al. (2024) [11] incorporated rGO into both self-cured and heat-cured acrylic resins. The objective of this study was to determine whether the addition of rGO could suppress microbial growth and biofilm development on polymer surfaces, focusing on bacteria frequently associated with oral infections. Acrylic resin specimens were produced with rGO loadings of 0% (control), 0.5%, 1%, and 3% (wt). These samples were challenged with three clinically relevant oral bacterial species, namely *Strep. mutans*, *Staphylococcus aureus* (*Staph. aureus*), and *Pseudomonas aeruginosa* (*P. aeruginosa*). The antifungal activity against *Candida albicans* (*C. albicans*) is discussed separately in Section 3.2. Antibacterial activity was assessed using three complementary analytical approaches: CFU quantification to evaluate microbial viability, XTT assays to measure metabolic activity, and scanning electron microscopy (SEM) to visualize microbial colonization on the resin surface. In acrylic resin composites containing rGO (0.5%, *w*/*w*), *Strep. mutans* viability was reduced compared with unmodified controls. In fact, CFU counts decreased from 6.69 ± 0.33 log CFU/mL in the control to 5.83 ± 0.57 log CFU/mL in the 0.5% (*w*/*w*) rGO self-cured resin, while metabolic activity assessed by the XTT assay declined from approximately 2.0 to 0.8 absorbance units at 490 nm (Table 7) [11]. These findings suggest that even low levels of rGO can impair *Strep. mutans* viability and disrupt its metabolic processes. *Staph. aureus* exhibited moderate reductions in growth and metabolic activity, although the results were less uniform across concentrations. In contrast, *P. aeruginosa* displayed no meaningful reduction in CFU or metabolic activity. At higher rGO concentrations (3%), the CFU counts of *P. aeruginosa* slightly increased to 8.45 ± 0.30 log_10_ CFU/mL in self-cured resin and 8.45 ± 0.21 log_10_ CFU/mL in heat-cured resin, indicating that rGO’s proposed antimicrobial mechanism, chiefly associated with oxidative damage and membrane perturbation, does not effectively target this highly resistant Gram-negative species (Table 7) [11].

These results indicate that rGO exhibits a selective antimicrobial profile, with its most pronounced activity directed against *Strep. mutans*, thereby reinforcing its potential as a functional component in caries-preventive dental materials. Nonetheless, the minimal impact observed against *P. aeruginosa* highlights an important limitation, suggesting that further refinement of rGO formulations, or their combination with additional antimicrobial agents, may be required to achieve a broader antimicrobial spectrum.

### 3.2. Antifungal Activity by Graphene-Based Materials

Acidogenic bacteria within oral biofilms, besides reducing the local pH and promoting the demineralization of enamel and dentin, as abovementioned, also favor the growth of fungi [63]. The antifungal potential of GO and rGO against *C. albicans* has been demonstrated across different experimental studies as outlined below.

#### 3.2.1. Enhancement in Antifungal Activity of rGO-Modified Acrylic Resins Against *C. albicans*

The antifungal potential of rGO as a nanofiller in dental polymers, incorporated into both self-cured and heat-cured acrylic resins, was also assessed [11]. This study aimed to determine whether the addition of rGO could suppress *C. albicans* growth and biofilm development on polymer surfaces often associated with oral infections. Acrylic resin specimens were produced with rGO loadings of 0% (control), 0.5%, 1%, and 3% (*w*/*w*) and *C. albicans* cells were exposed to the material samples. Antifungal activity was assessed using three complementary analytical approaches: CFU quantification to evaluate microbial viability, XTT assays to measure metabolic activity, and scanning electron microscopy (SEM) to visualize microbial colonization on the resin surface. In acrylic resin composites containing rGO (0.5%, *w*/*w*), *C. albicans* exhibited moderate reductions in growth and metabolic activity, although the results were less uniform across concentrations (Table 7). Future studies with different resin types should be carried out to assess whether antifungal activity depends on the resin type.

#### 3.2.2. Enhancement in Antifungal Activity of GNP-Modified PMMA Against *C. albicans*

Salgado et al. (2022) [1] expanded their analysis to encompass the antifungal efficacy of PMMA modified with GNPs, focusing on *C. albicans*, a principal etiological agent of denture-related infections such as denture-related stomatitis. For this purpose, *C. albicans* was exposed to 3D-printed PMMA specimens containing GNPs at concentrations of 0.01%, 0.1%, 0.25%, and 0.5% (wt) for 24 and 48 h. Across all tested concentrations and time points, a reduction in *C. albicans* biofilm formation to approximately 1.08–1.67 log CFU/mL was observed relative to the control group (1.77 log CFU/mL). Notably, all tested concentrations of GNPs exhibited similar antifungal activity against *C. albicans*, with no statistically significant differences detected among them. This suggests that increasing the graphene content does not further enhance the inhibitory effect. Consequently, even low GNP concentrations appear sufficient to achieve effective antifungal performance, allowing for the potential minimization of graphene loading, which may help minimize potential cytotoxicity while maintaining antimicrobial efficacy [1]. These results indicate that the antifungal activity of GNPs was consistent across the tested concentrations, underscoring their potential to limit fungal colonization on prosthetic surfaces.

#### 3.2.3. Enhancement in Fluconazole Antifungal Activity Against *C. albicans* Through the Combined Application with GO

Azole-class antifungals, particularly fluconazole (Flu), remain the primary therapeutic agents for managing *Candida* infections. Nonetheless, the increasing prevalence of resistance, particularly in *C. albicans*, poses a significant clinical challenge. Such resistance is predominantly associated with either overexpression or point mutations in ERG11 gene, encoding lanosterol 14α-demethylase, the enzymatic target of azoles. Overexpression elevates the quantity of the target enzyme, whereas mutations modify their structural conformation. Both events diminish Flu’s binding affinity and consequently impair its antifungal efficacy. In response to this growing resistance, recent research [50,52] has examined the synergistic application of GO with Flu. GO possesses inherent antifungal capabilities, including disruption of cell membranes, induction of oxidative stress, and inhibition of biofilm formation. When combined with Flu, GO may enhance intracellular drug uptake and potentially restore susceptibility in fluconazole-resistant strains. One such study [64] demonstrated that a GO/Flu nanocomposite markedly reduced the viability of a fluconazole-resistant *C. albicans* strain (ATCC 10231), outperforming Flu alone. These results highlight GO’s role as an effective sensitizing agent and emphasize its potential in overcoming azole-resistant fungal infections. The same study also assessed the release profile of Flu from the GO/Flu nanocomposite. In phosphate-buffered saline (PBS, pH 7.4), a standard medium for simulating physiological conditions, the nanocomposite released Flu more slowly and in a more controlled manner than Flu alone. This prolonged release profile is advantageous, as it maintains the minimal therapeutic intracellular concentration required for synergistic antifungal action while reducing the likelihood of human cytotoxicity. Human cytotoxicity was evaluated using MTT assays, which revealed that GO/Flu displayed a dose-dependent reduction in viability of the human SW480 colorectal cell line but exhibited significantly lower toxicity compared with GO or Flu administered individually. This is noteworthy given that GO alone was more cytotoxic than Flu. Furthermore, adhesion assays showed that the GO/Flu nanocomposite substantially diminished the capacity of *C. albicans* to adhere to SW480 cells, outperforming either with Flu or GO alone. This suggests that the nanocomposite not only reduces fungal survival but also impairs early colonization mechanisms. Complementary DNA fragmentation assays revealed that GO/Flu induced markedly greater DNA degradation in *C. albicans* than any of the individual treatments or the untreated control, supporting the contribution of DNA damage to the observed antifungal activity [64]. Collectively, these findings demonstrate that the GO/Flu nanocomposite exhibits potent anti-*Candida* activity with minimal cytotoxicity toward mammalian cells, supporting its potential value as a therapeutic platform and justifying further in vivo investigation to validate its safety and efficacy. Although this study evaluated the nanocomposite in its isolated form, without incorporation into a polymeric matrix, the results carry strong implications for dental materials research. GO/Flu nanocomposites may represent a potential strategy for future incorporation into dental polymer matrices into widely used dental polymers, including hydrogels, acrylic resins, or soft lining materials, to confer targeted antifungal properties in clinical applications. This strategy may be particularly advantageous for the prevention of denture-related stomatitis or for managing patients susceptible to recurrent oral candidiasis, offering a promising route for translating nanotechnology into advanced polymer-based dental biomaterials.

### 3.3. Safety Considerations of Graphene-Based Materials: Human Cytotoxicity, Biological Interactions and Biocompatibility

**Figure 3:**
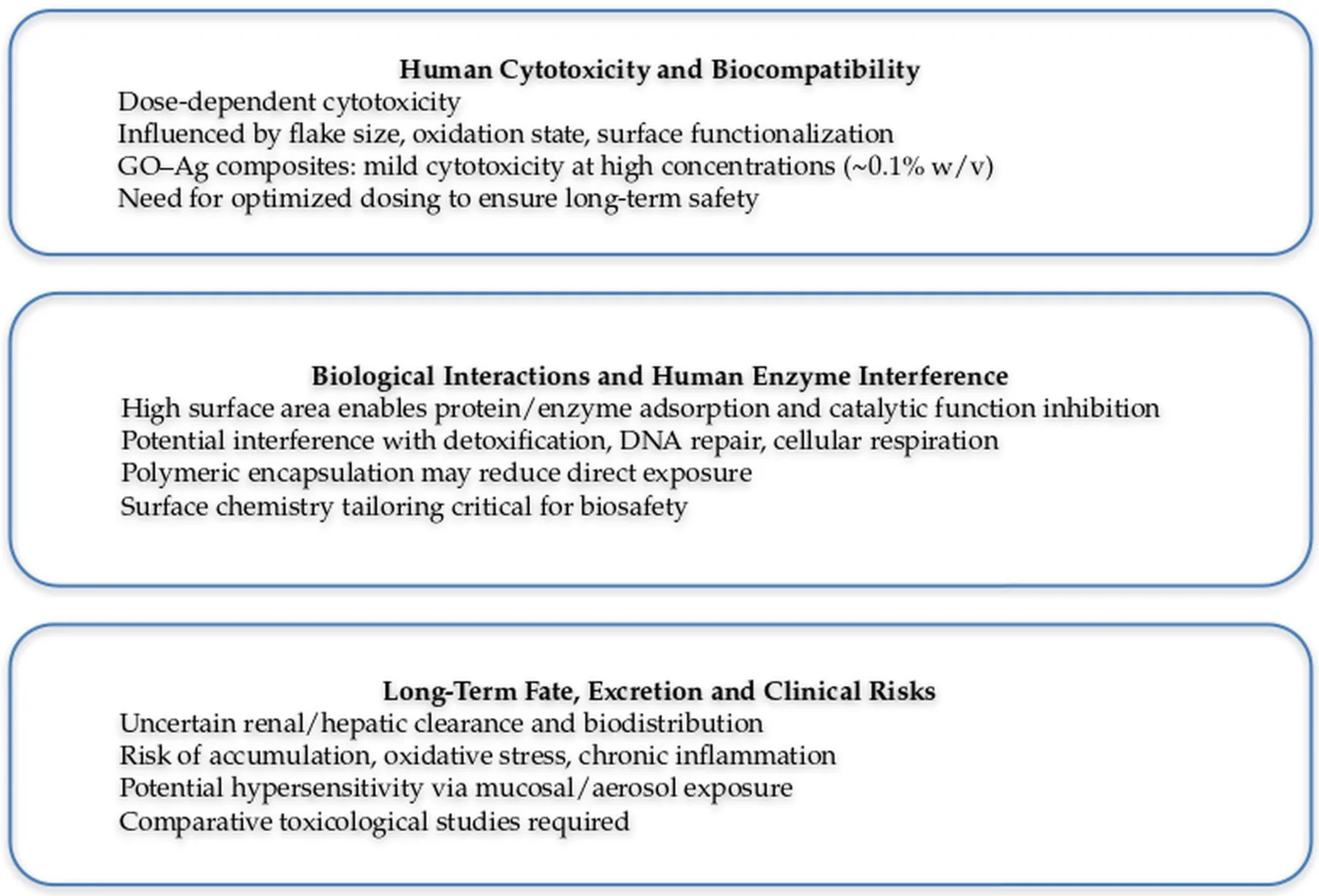
Safety considerations of graphene-based materials in dental applications, highlighting dose-dependent cytotoxicity, enzyme interactions, and uncertainties regarding long-term biodistribution and clinical risks.

Reported in vitro biocompatibility of GO, rGO, and GNPs, combined with their broad-spectrum antimicrobial activity and highly tunable physicochemical properties, including modifiable surface chemistry, adjustable oxidation states, and relatively high surface area, supports their integration into polymer-based drug delivery systems, wound-healing scaffolds, and dental tissue engineering materials [30,51,65,66]. It should be highlighted that the properties and biological effects of GO, rGO, and GNPs can vary substantially depending on synthesis method, degree of oxidation or reduction, flake size, and surface functionalization, and conclusions should be interpreted considering the characterization reported in each study (Figure 3).

While graphene-based materials exhibit considerable antibacterial and antifungal potential in dental polymeric materials, their biological interactions during clinical use warrant careful consideration. Evidence from recent studies indicates that these materials may present occupational and handling hazards due to potential occupational exposure risks for dental professionals and cytotoxic effects in patients exposed to dental devices containing graphene-based materials. The extent of toxicity/cytotoxicity is influenced by multiple factors, including the size and morphology of graphene nanoflakes, concentration, surface area, type of graphene, functionalization strategy, electrical conductivity, duration of exposure, regulation of oxidative stress, exposure to thermal or photonic irradiation, and overall biocompatibility with living tissues [22,23,31]. Consequently, assessment of the optimal dosing parameters is essential to ensure long-term biocompatibility, minimize cytotoxic risks, and support tissue regeneration.

GO, rGO, and GNPs have emerged as promising materials in biomedical research due to their antimicrobial activity against bacteria and fungi.

GO is commonly incorporated into natural and synthetic polymer matrices such as Poly(lactic-co-glycolic acid) (PLGA) and PVA, where it improves mechanical strength, modulates degradation profiles, and enables controlled drug release. In dentistry, GO–polymer composites are being explored for antimicrobial coatings, restorative materials, and thin-film barriers, benefitting from the synergistic bioactivity of GO and the structural versatility of polymers [50,51,65]. The cytotoxicity of functionalized GO formulations shown in Table 2 was assessed by MTT assays using human epithelial cells across a range of concentrations. At lower doses, all formulations exhibited negligible cytotoxicity, remaining within acceptable biocompatibility limits. At the highest concentration tested (0.1% *w*/*v*), mild cytotoxic effects were observed for GO–Ag and GO–Ag–CaF_2_, namely GO–Ag–CaF_2_ reduced cell viability to ~85% compared with control [7]. These findings indicate that GO functionalized composites are generally biocompatible at clinically relevant concentrations, although concentration-dependent cytotoxic responses should be taken into account when considering their application to epithelial tissues.

rGO, with its partially restored graphitic structure and reduced oxygen content, provides enhanced electrical conductivity and mechanical stability relative to GO. Although effective in polymeric systems, achieving antimicrobial activity often requires higher concentrations or specific functionalization strategies, necessitating careful human cytotoxicity assessment. rGO is especially relevant for applications that prioritize electrical functionality or reinforcement, including electroactive scaffolds and implant coatings [30,53,67].

GNPs, composed of multiple stacked graphene layers, are increasingly utilized to reinforce dental polymer matrices. Although less oxidized and therefore typically less antimicrobial than GO, GNPs possess moderate bioactivity that can be augmented through surface modification or co-delivery with other antimicrobial agents. Their compatibility with diverse polymer systems and their capacity to enhance thermal and structural stability further support their application in dental nanocomposites [52,53].

#### 3.3.1. GO, rGO, and GNPs Interactions with Human Enzymes

A critical emerging concern regarding the use of GO, rGO, and GNPs in dental medical applications is their potential to interfere with human enzymatic activity. Due to their relatively high surface area and reactive chemistries, graphene-based materials may adsorb or bind enzymes essential not only for microbial metabolism but also for human physiological processes, potentially altering catalytic function [30,50,64]. In humans, unwanted interactions of GO, rGO, and GNPs with enzymes involved in cellular detoxification, DNA repair, or cellular respiration could elicit adverse biological responses. GO may sequester cofactors or compete for active sites, while rGO and GNPs may produce similar, albeit typically weaker, effects due to their lower oxygen content [30,50,64]. Polymeric encapsulation may mitigate enzyme interference by limiting direct exposure to biological fluids. Tailoring surface chemistry and composite design parameters, such as graphene loading, dispersion state, interfacial adhesion, and polymer matrix physicochemical properties, is therefore essential to optimize biosafety [30,50,65].

#### 3.3.2. GO, rGO, and GNPs Excretion and Long-Term Biological Fate

The excretion pathways and long-term biodistribution of GO, rGO, and GNPs remain insufficiently characterized. Many studies do not conclusively determine whether these materials are effectively cleared via renal or hepatic routes or whether they accumulate in tissues over time. Persistent accumulation may be associated with chronic inflammation, organ toxicity, or immune dysregulation [30,50,65]. These concerns are particularly pertinent to dental applications, where exposure may be continuous or repeated, such as implant coatings, denture liners, or polymeric restorative materials. GO did not affect red blood cell morphology at 6.25 μg/mL [58]. However, GO and rGO, due to their nanoscale dimensions and surface reactivity, may promote oxidative stress or inflammatory responses in adjacent tissues [50,51,65]. GNPs, although less likely to penetrate deeply in tissues, still require long-term safety evaluation [52,53].

Potential hypersensitivity reactions, especially through mucosal exposure or aerosolized particles during clinical handling, must also be considered. Comparative toxicological studies across graphene-based materials are needed to clarify their immunological profiles and the risk of accumulation in vital organs [30,50,64].

## 4. Conclusions and Future Perspectives

Notwithstanding the extensive investigation of graphene-based nanomaterials, a comprehensive understanding and comparison of their antimicrobial performance in polymeric matrices remain limited and fragmented. In addition, the wide diversity of synthesis methods, physicochemical properties, and experimental conditions further complicates the interpretation and clinical translation of existing findings, particularly in dental polymer applications. This review aimed to provide a systematic and critical evaluation of current knowledge on the physicochemical, antibacterial, and antifungal properties of GO, rGO, and GNPs, their mechanisms of action, and their interactions with biological systems. It further identifies the main limitations affecting the integration of these materials into polymer-based dental systems, emphasizing the necessity of addressing these challenges to optimize graphene-based materials for dental applications and advance this field of research.

Recent evidence highlights the substantial potential of graphene-based materials, particularly GO, and, to a lesser extent, rGO and GNPs for antimicrobial applications in dental practice, owing to their distinctive structural and physicochemical characteristics. Among these materials, GO stands out because of its high hydrophilicity, ease of surface functionalization, and relatively favorable biocompatibility, positioning it as a particularly promising candidate for dental applications. The incorporation of these graphene-based materials into natural and synthetic polymer matrices has emerged as an effective strategy for enhancing material performance. For example, GO–polymer composites have demonstrated improved mechanical strength, controlled degradation behavior, enhanced biological responses, and potential for regulated drug delivery. Similarly, rGO and GNPs, especially when functionalized or integrated into hybrid systems, can reinforce structural properties and impart antimicrobial functionality, although achieving effects comparable to GO often requires higher loadings or additional chemical modification. It should be noted that the physicochemical properties and biological effects of GO, rGO, and GNPs can vary substantially depending on synthesis methods, degree of oxidation or reduction, flake size, and surface functionalization. Consequently, interpretation of their antibacterial and antifungal efficacy and biosafety must be closely linked to comprehensive material characterization. Surface chemistry, including electron donor–acceptor (EDA) interactions, further modulates antimicrobial and photothermal activity, underscoring the need for rational material selection and optimization in dental polymer systems.

Despite significant progress, several critical challenges remain, particularly with respect to safety and long-term biological performance. Potential interference with human enzymatic processes, uncertainties regarding chronic biocompatibility, immune responses, and the accumulation or clearance of graphene-based materials in biological tissues require thorough investigation. These issues are especially relevant in complex environments such as the oral cavity, where fluctuating pH, mechanical loading and complex microbial communities may influence material behavior. Future research should therefore focus on elucidating the in vivo interactions of GO, rGO, and GNPs, both when used alone or within polymeric dental composites, with particular emphasis on structure–activity relationships, degradation pathways, and host–material interfaces. A comprehensive understanding of long-term safety, tissue integration, and immunological responses under physiologically relevant oral conditions is essential for successful clinical translation of GO, rGO, and GNPs into dental clinical practice. Moreover, selective antibacterial and antifungal activity must be carefully balanced against potential effects on host cells, enzymatic processes, and tissue function. Considering the distinct properties of each graphene-based material, namely GO’s high reactivity and surface chemistry versatility, rGO’s enhanced conductivity and structural stability, and GNP’s mechanical reinforcement potential, material designs should be tailored to specific dental applications. The use of polymer matrices to regulate interfacial interactions represents a promising approach to maximizing therapeutic efficacy while minimizing biosafety risks. In parallel, emerging technological advances, including additive manufacturing techniques for dental polymers (such as 3D printing of dental polymers) and data-driven methodologies incorporating artificial intelligence, may facilitate the rational design, optimization, and scalable production of graphene-based dental materials. These tools offer new opportunities to accelerate the translation of laboratory findings into clinically relevant products.

Beyond graphene-based materials, graphitic carbon nitride (g-C_3_N_4_) has emerged as a distinct carbon- and nitrogen-based nanomaterial with potential antimicrobial applications. Recent studies have reported antibacterial activity associated with photocatalytic and reactive oxygen species-mediated mechanisms, while emerging research has also explored its potential relevance to oral healthcare and dental materials [68,69,70]. Nevertheless, its antimicrobial performance and biocompatibility depend on material composition, surface modification, illumination conditions, and experimental models. Further studies are required to establish its efficacy against clinically relevant oral microorganisms, assess its compatibility with dental polymer matrices, and characterize its cytotoxicity and long-term biological safety. As g-C_3_N_4_ is structurally distinct from GO, rGO, and GNPs, it remains outside the comparative scope of the present review but represents a promising direction for future research in dental biomaterials.

In conclusion, among the graphene-based materials reviewed, GO appears to be the most promising candidate for antimicrobial dental applications, owing to its hydrophilicity, ease of surface functionalization, and reported antibacterial and antifungal potential. However, this advantage should not be generalized, as antimicrobial performance depends on the material’s physicochemical characteristics, functionalization, concentration, dispersion within the polymer matrix, and the microorganisms and experimental conditions tested. Although rGO and GNPs have also shown potential in specific dental polymer formulations, further comparative studies under simulated intraoral conditions and in vivo are needed to establish their relative efficacy, safety, and clinical relevance. Successful translation will require balancing antimicrobial performance with material properties, aesthetic outcomes, and long-term biocompatibility.

## Figures and Tables

**Table 1:** Qualitative comparison of general physicochemical properties of graphene oxide (GO), reduced graphene oxide (rGO), and graphene nanoplatelets (GNPs). Data on oxygen content, aqueous dispersibility, surface area, and electrical conductivity are from [28,29,45,46,49]. The qualitative descriptors (High/Moderate/Low) reflect commonly reported trends of the material’s properties and do not replace material-specific characterization by techniques such as XPS or FTIR.

Material			Properties ^1^	
	Oxygen Content	Aqueous Dispersibility	Surface Area	Electrical Conductivity
GO	High	High	High	Low
rGO	Moderate	Moderate	Moderate	High
GNPs	Low	Low	High	High

^1^ The quality descriptors of the properties reported in this table may vary significantly depending on synthesis method, degree of oxidation or reduction, lateral size, thickness, and surface functionalization. Accurate identification of graphene-based materials requires material-specific characterization (e.g., XPS, FTIR).

**Table 2:** XPS-Characterized Surface Chemistry of Graphene-Based Materials and Their Biological Implications. Data from [16,24,25,26,27,30].

Material	Surface Chemistry Features (XPS)	Dominant Functional Groups	Biological Relevance
GO	High O/C ratio; strong C–O and O–C=O peaks; increased sp^3^ carbon contribution	Hydroxyl (C–OH), epoxy (C–O–C), carbonyl (C=O), carboxyl (O–C=O)	Increased hydrophilicity and negative surface charge Enhanced protein adsorption Stronger interaction with microbial membranes Contribution to oxidative stress mechanisms
rGO	Lower O/C ratio compared with GO; partial restoration of sp^2^ carbon network; residual oxygen peaks	Residual hydroxyl and carbonyl groups; increased graphitic C–C/C=C	Higher electrical conductivity Increased electron transfer capacity Potential ROS-mediated antimicrobial effects Altered protein corona formation
GNPs	Predominantly sp^2^ carbon; minimal oxygen signal; strong graphitic C–C peak	Mainly graphitic carbon with minor edge oxygen groups	Lower dispersibility in aqueous media Reduced protein adsorption Generally lower biological reactivity compared with GO Interaction dominated by π–π and hydrophobic forces

**Table 3:** Comparison of antibacterial performance of GO-based composites against *Strep. mutans*, based on estimates of CFU percentage reduction obtained from graphical representations reported [7,59]. The antibacterial agent povidone-iodine and growth medium used as a positive and negative control, respectively.

Material/Composite	Concentration (%, *w*/*v*)	Antibacterial Activity	Key Observation
Povidone-iodine	0.1	Very high	Positive control
GO	0.01–0.1	Moderate	Concentration-dependent
GO–Ag	0.01–0.1	Very high	Near-complete CFU (%) reduction
GO–Ag–CaF_2_	0.01–0.1	Very high	Synergistic antibacterial effect
GO–CaF_2_	0.01–0.1	Low-Moderate	Minimal CFU (%) reduction
GO–Zn	0.01–0.1	Low	Minor CFU (%) reduction
GO–Ca_3_(PO_4_)_2_	0.01–0.1	Minimal	No meaningful CFU (%) reduction
Growth medium	—	None	Negative control

**Table 4:** Comparison of antibacterial performance of GO-based composites against *Strep. mutans*, based on the reduction in bacterial metabolic activity following exposure to GO-based nanocomposites at different concentrations. adapted from [7]. Metabolic activity was assessed by XXT assay. Qualitative descriptors of metabolic activity and antimicrobial effect are intended for comparative purposes of antibacterial efficacy.

Material System	Concentration (%, *w*/*v*)	Metabolic Activity (Absorbance at 490 nm) *	Antimicrobial Effect
Control (Medium)	—	High (~2.0)	None
Povidone–iodine	0.1	Very low (~0.2)	Strong
GO	0.01–0.1	High (~1.9–2.0)	Weak
GO–Ag	0.01–0.1	Moderate-high (~1.3–1.8)	Weak-moderate
GO–Ag–CaF_2_	0.01–0.1	Low-moderate (~0.5–1.5)	Moderate-strong
GO–CaF_2_	0.01–0.1	High (~2.0)	Weak
GO–Zn	0.01–0.1	Moderate-low (~0.9–1.8)	Moderate
GO–Ca_3_(PO_4_)_2_	0.01–0.1	Moderate-high (~1.7–1.8)	Weak-moderate

* The estimative absorbance values at 490 nm were obtained from graphical data reported by [7].

**Table 5:** Antibacterial activity of GNPs-enhanced PMMA against *Strept. mutans* at 24h and 48h incubation times, expressed as log CFU/ mL. Adapted from [1]. A control without graphene (Ct) and a control with pure graphene (Gc) were used.

Graphene/GNPs Concentration (%, wt)	0 h (log CFU/mL)	24 h (log CFU/mL)	48 h (log CFU/mL)
Ct	~9.5	~12.2	~12.8
Gc	~9.6	~12.4	~12.7
GNPs 0.01	~9.5	~10.7	~7.8
GNPs 0.10	~9.7	~10.4	~8.2
GNPs 0.25	~9.6	~10.2	~7.9
GNPs 0.50	~9.7	~9.8	~7.8

**Table 6:** Surface roughness (Ra) of 3D-printed PMMA resin as a function of GNP loading. Data adapted from [1].

Sample	Graphene Loading (%, *w*/*w*)	Ra (µm) (Mean ± SD)	Statistical Significance *
Gc	0.00	~1.10 ± 0.15	–
GNPs0.01	0.01	~1.05 ± 0.15	ns
GNPs0.1	0.10	~1.25 ± 0.18	ns
GNPs0.25	0.25	~1.80 ± 0.20	*
GNPs0.5	0.50	~1.90 ± 0.18	*

* Statistical significance relative to the graphene control (Gc): *p* < 0.05; ns, not statistically significant.

**Table 7:** Summary of antimicrobial effects rGO-incorporated acrylic resins against oral pathogens, based on cell viability and metabolic activity (XTT) assays. Trends are qualitative in comparison with the negative controls in the absence of the rGO-incorporated acrylic resins. Qualitative descriptors were driven from the experimental data [11].

Oral Pathogens	Cell Viability (CFU Assays)	Metabolic Activity (XTT Assays)	Implications for Dental Materials
*Strep. Mutans*	Reduced	Reduced	rGO may be effective for caries-preventive resins
*Staph. Aureus*	No significant reduction	Slightly reduced	Possible partial inhibition
*C. albicans*	No significant reduction	Slightly reduced	Activity may depend on resin type
*P. aeruginosa*	No reduction/slight increase	No reduction	rGO not effective against this strain

## Data Availability

No new data were created or analyzed in this study. Data sharing is not applicable to this article.

## Abbreviations

The following abbreviations are used in this manuscript:

*C. albicans*	*Candida albicans*
CFU	Colony-forming unit
CS	Chitosan
EDTA	Ethylenediaminetetraacetic acid
Flu	Fluconazole
GNPs	Graphene nanoplatelets
GNRs	Graphene nanoribbons
GO	Graphene oxide
GOns	Graphene oxide nanosheets
GQDs	Graphene quantum dots
HSV-1	Herpes simplex virus Type-1
*P. aeruginosa*	*Pseudomonas aeruginosa*
PEG	Polyethylene glycol
PLGA	Poly(lactic-co-glycolic acid)
PMMA	Polymethyl Methacrylate
PVA	Polyvinyl alcohol
RBCs	Red blood cells
rGO	Reduced graphene oxide
ROS	Reactive oxygen species
*Staph. aureus*	*Staphylococcus aureus*
*Strep. mutans*	*Streptococcus mutans*
SEM	Scanning electron microscopy
SMRGO	Sulfonated magnetic nanoparticles with reduced graphene oxide
TA	Tannic Acid
XPS	X-ray photoelectron spectroscopy
ZnO	ZnO
ZnO NPs	Zinc Oxide Nanoparticles
